# Identification of biomarkers, pathways and potential therapeutic target for docetaxel resistant prostate cancer

**DOI:** 10.1080/21655979.2021.1936831

**Published:** 2021-06-02

**Authors:** Rui-ji Liu, Shu-ying- Li, Li-quan Liu, Bin Xu, Ming Chen

**Affiliations:** a Department of Urology, Affiliated Zhongda Hospital of Southeast University, Nanjing, China; b Surgical Research Center, Institute of Urology, Southeast University Medical School, Nanjing, China; c Sichuan Cancer Hospital & Institute, Sichuan Cancer Center, Cancer Hospital Affiliate to School of Medicine, UESTC, Chengdu, China; d Department of Urology, Meishan City People’s Hospital, Meishan, China; e Nanjing Lishui District People’s Hospital, Zhongda Hospital Lishui Branch, Southeast University, Nanjing, China

**Keywords:** CRPC, docetaxel resistance, prognosis, GEO

## Abstract

Docetaxel has been proved to provide survival benefit for advanced prostate cancer (PCa) patients. Resistance to docetaxel further reduces the survival of these patients. Herein, we performed a comprehensive bioinformatic analysis to identify differentially expressed genes (DEGs) between docetaxel sensitive and resistant PCa (DRPC) cell based on Gene Expression Omnibus (GEO) database. Gene Ontology (GO) and Kyoto Encyclopedia of Genes and Genomes (KEGG) analyses were applied for functional and pathway analysis of DEGs. The STRING database, cytoscape software and plug-in ‘cytoHubba’ were used to construct protein–protein interaction (PPI) networks and identify hub genes. Survival analysis were performed via GEPIA database. Finally, we conducted immune infiltration analysis by TIMER. A total of 460 DEGs were identified. GO functional analysis showed that these DEGs are mainly enriched in chemotaxis, negative regulation of intracellular signal transduction, and regulation of cell adhesion, positive regulation of inflammatory response, regulation of response to cytokine stimulus. According to the results of KEGG pathway analysis, these DEGs are mainly involved in signaling by Rho GTPases, Miro GTPases and RHOBTB3; interferon Signaling; arginine biosynthesis; PI3K-Akt signaling pathway; cytokine-cytokine receptor interaction; MAPK signaling pathway. Finally, CCNB1 and EZH2 were identified as prognostic hub genes and the expression of these two genes were associated with immune infiltration. The present study may helps to improve the understanding of the molecular mechanisms of DRPC and facilitate the selection of therapeutic and prognostic biomarkers for DRPC.

## 1. Introduction

Prostate cancer (PCa) is one of the most common malignancies and remains the second most deadly disease in men worldwide [1]. It is well known that the growth and progression of PCa is dependent on androgens, and androgen deprivation therapy (ADT), whether chemical or surgical deprivation, can achieve good results in the early stages of treatment [2–4]. However, due to multiple molecular mechanisms leading to reactivation of the androgen receptor (AR) signaling pathway, such as AR mutations, AR overexpression/amplification, and AR splicing variants, castration resistant prostate cancer (CRPC) is inevitable after approximately 2 years of treatment [5–8]. Compared to early, localized cases, CRPC has much shorter of median survival time and poorer quality of life [9,10].

Chemotherapy can reduce serum prostate-specific-antigen (PSA) levels in PCa patients, and in some cases can reduce pain [11]. Docetaxel is an m-phase cycle specific drug that promotes tubules aggregation to form stable microtubules and inhibits their depolymerization by binding β-tubulin, thereby significantly reducing the number of tubules and destroying the microtubule reticular structure [12]. Two prospective phase III trials (The TAX 327 and SWOG 99–16), had demonstrated a survival benefit of docetaxel in patients with CRPC patients, with a median survival benefit approximately 2.5-months [11,13]. In 2004, the US FDA approved docetaxel as a new standard treatment for metastasis CRPC. However, some cases initially respond poorly to docetaxel-based therapy, and eventually all patients will develop docetaxel-resistance. Therefore, understanding the molecular mechanisms underlying the development of DRPC and selection of new biomarkers can help identify new therapeutic targets to prolong the survival and improve the quality of life of DRPC patients.

Currently, microarrays and bioinformatics analysis are being used to screen for differentially expressed genes (DEGs) in tumorigenesis and epigenetic variations. Researchers can access high-throughput microarray and next-generation sequence functional genomic data from the international public repository Gene Expression Omnibus (GEO), and download them for free [14]. Multiple biomarkers are highly expressed in PCa, but the specific markers in DRPC remains unknown.

In this study, we conducted comprehensive bioinformatic analyses to identify prognostic hub genes in DRPC. DEGs between docetaxel sensitive and resistant prostate cancer cell were identified using GSE33455 and GSE36135 [15,16]. Protein–protein interaction (PPI) network and survival analyses were performed. Finally, CCNB1 and EZH2 were identified as prognostic hub genes in DRPC. These findings may contribute to the understanding of the molecular mechanism underlying the development of DRPC and provide new gene targets for future studies.

## 2. Methods

### 2.1 Data acquisition and identification of DEGs

After a systematic search, GSE33455 [15] and GSE36135 [16] were finally included and downloaded from GEO database (http://www.ncbi.nlm.nih.gov/geo/). The detailed information of these two datasets were summarized in Table 1. Gene expression data of GSE33455 and GSE36135 were analyzed using GEO2R, with significant cutoff value setting at |log_2_ FC | > 1, adjust *P* value < 0.05 using Benjamini & Hochberg correction [14].

**Table 1.** The detailed information of the two datasets

Dataset	Number of samples	Array types	Cell lines
	(Sensitive/Resistant)		
GSE33455 [15]	6-Jun	GPL570 platform (Affymetrix Human Genome U133 Plus 2.0 Array)	PC3, Du145
GSE36135 [16]	6-Jun	GPL570 platform (Affymetrix Human Genome U133 Plus 2.0 Array) and GPL571 platform (Affymetrix Human Genome U133A 2.0 Array)	22 Rv1, Du145

### 2.2 GO analysis and KEGG pathway analysis

GO analysis is widely used for gene functional classification and gene annotation including biological process (BP), cellular component (CC), and molecular function (MF) [17]. KEGG is a database resource that integrates genomic, chemical, and systemic functional information [18,19]. GO functional enrichment and KEGG pathway analysis of DEGs were investigated through the Metascape (https://metascape.org/) [20].

### 2.3 PPI network construction

All DEGs were imported to the construction of protein-protein interaction network using the Search Tool for the Retrieval of Interacting Genes (STRING) (https://string-db.org/) with minimum required interaction score > 0.7 [21]. Non-interacting DEGs were removed. Cytoscape (version 3.6.1) and plug-in ‘cytoHubba’ were utilized for hub gene identification (The top 10 genes obtained) [22,23].

### 2.4 Expression and survival analyses of hub genes in PCa

RNA-seq data of PRAD dataset were obtained from TCGA (https://portal.gdc.cancer.gov/). We compared the expression of the hub genes in PCa and normal tissues with Wilcoxon rank sum test. Gene Expression Profile Interactive analysis (GEPIA) (http://gepia.cancer-pku.cn/) is a newly developed web server based on the TCGA and the GTEx projects which contains 9,736 tumor and 8,587 normal samples of RNA sequencing expression data [24]. In this study, the DFS analysis for expression of hub genes between high- and low expression group was performed based on PRAD dataset, and the log-rank test *P* value < 0.05 was considered statistically significant.

### 2.5 Immunohistochemistry analyses of prognostic hub genes

Human Protein Atlas database (https://www.proteinatlas.org/) was used for validation the expression of prognostic hub genes in protein level.

### 2.6 Correlation between immune infiltration and expression of EZH2 and CCNB1 in PCa

To further investigate the association between immune infiltration and the expression of EZH2 and CCNB1, we used TIMER web server (https://cistrome.shinyapps.io/timer/), which is a comprehensive resource for systematical analysis of immune infiltrates across diverse cancer types [25].

### 2.7 Software and versions

R software (x64, version 4.0.3) was used for statistical calculations and graphs (https://www.r-project.org/).

## 3. Results

In this work, we performed DEGs analysis between docetaxel resistant and sensitive PCa cells and confirmed the functions and pathways of these DEGs. Finally, we identified CCNB1 and EZH2 as prognostic hub genes in docetaxel resistant PCa and further investigated the relationship between immune infiltration and these two genes.

### 3.1 Identification of DEGs

**Figure 1.**
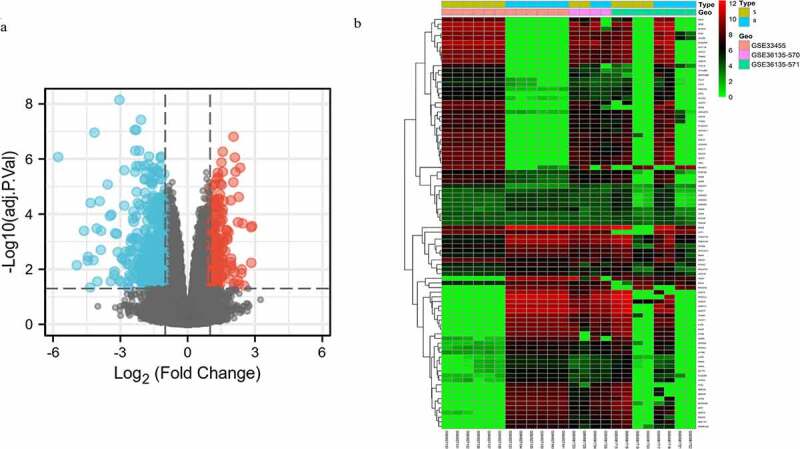
Volcano plot distribution and heatmap of the DEGs. (a) Volcano plot of GSE33455 and GSE36135. The red points indicate upregulated DEGs, the blue points indicate downregulated DEGs, and the gray points indicate DEGs with no significant difference in expression; (b) DEG heatmap of GSE33455 and GSE36135. From red to green, the expression level of the genes in the samples gradually decreases. All DEGs are screened based on adj. *P* value < 0.05, |log_2_ FC | > 1. (DEGs, differentially expressed genes; S, docetaxel-sensitive; R, docetaxel-resistant)

A total of 460 DEGs were identified based on GSE33455 and GSE36135, with |log_2_ FC | >1, adjust *P* value <0.05. Volcano plot distribution map and cluster heatmap of these DEGs were shown in Figure 1(a, b).

### 3.2 GO analysis and KEGG pathway analysis

**Figure 2.**
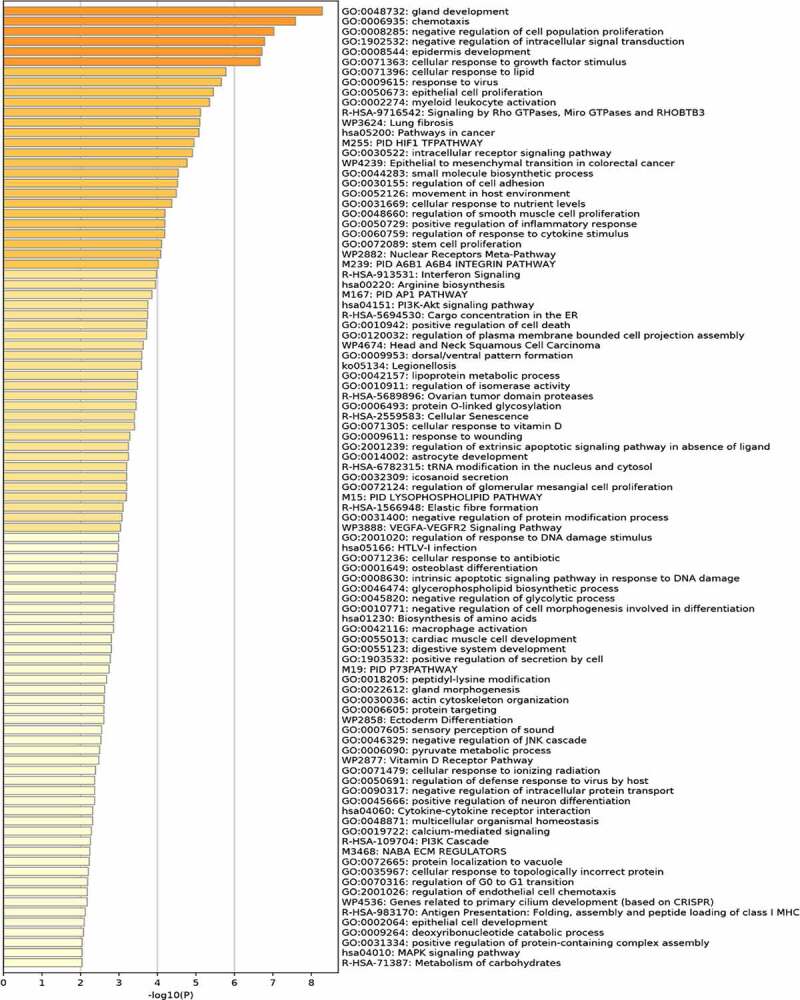
Functional enrichment analysis of DEGs

GO functional analysis of these DEGs showed DEGs were mainly enriched in chemotaxis, negative regulation of cell population proliferation, negative regulation of intracellular signal transduction, cellular response to growth factor stimulus, cellular response to lipid, intracellular receptor signaling pathway, regulation of cell adhesion, positive regulation of inflammatory response, regulation of response to cytokine stimulus, lipoprotein metabolic process. Barplot of GO enrichment analysis was shown in Figure 2 (*P* < 0.05).

KEGG pathway analysis showed that these DEGs were mainly involved in the signaling by Rho GTPases, Miro GTPases and RHOBTB3, interferon Signaling, arginine biosynthesis, PI3K-Akt signaling pathway, cytokine-cytokine receptor interaction, MAPK signaling pathway. Barplot of KEGG analysis was shown in Figure 2 (*P* < 0.05).

### 3.3 PPI network construction

**Figure 3.**
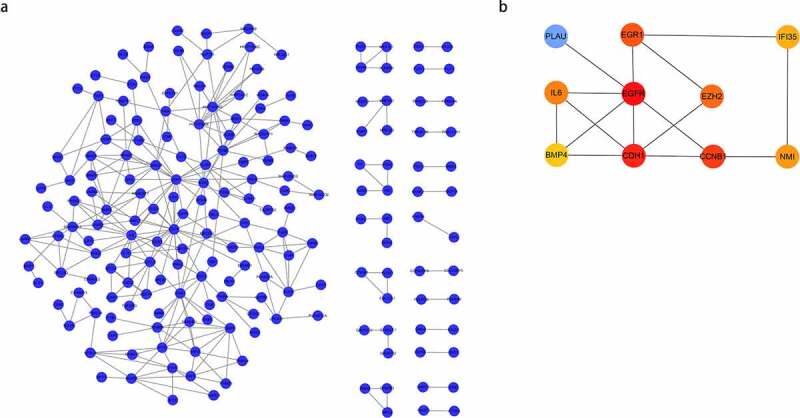
(a) Protein-Protein Interaction (PPI) network of differentially expressed genes (DEGs); (b) hub gene network construction using ‘cytoHubba.’

All DEGs were imported into STRING to construct the protein-protein interaction network. Cytoscape (version 3.6.1) was applied for visualization of the network, and plug-in ‘cytoHubba’ was utilized for hub gene network construction. Finally, PLAU, EGR1, IFI35, IL6, EGFR, EZH2, BMP4, CDH1, CCNB1, and NMI were obtained (Figure 3).

### 3.4 Expression and survival analyses of hub genes in PCa

**Figure 4.**
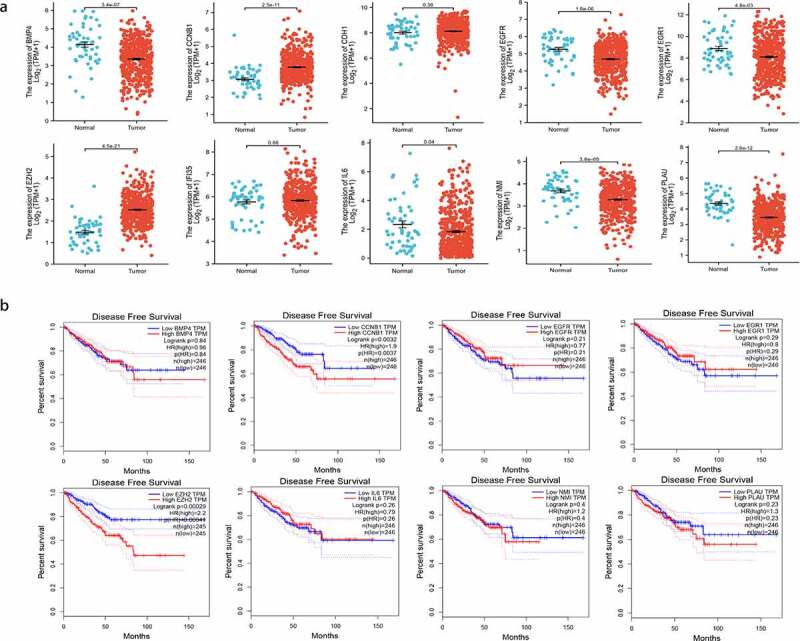
(a) Gene expression of the 10 hub genes between tumor and normal tissues; (b) Disease-free survival (DFS) analysis of the hub genes (*P* < 0.05)

The expression of these ten hub genes in PCa and normal tissues were shown in Figure 4a. The difference in expression comparing tumor and normal tissues were found to be significant in eight genes (PLAU, EGR1, IL6, EGFR, EZH2, BMP4, CCNB1, NMI). In order to investigate the correlation between the expression of these genes and prognosis in PCa patients, we performed survival analysis using GEPIA based on prostate adenocarcinoma (PRAD) dataset. Finally, EZH2 and CCNB1 were found to be associated with prognosis (Figure 4b). High expression of EZH2 (HR = 2.2, *P* = 0.00041), CCNB1 (HR = 1.9, *P* = 0. 0037) were associated with poorer DFS.

### 3.5 Immunohistochemistry analyses of prognostic hub genes

**Figure 5.**
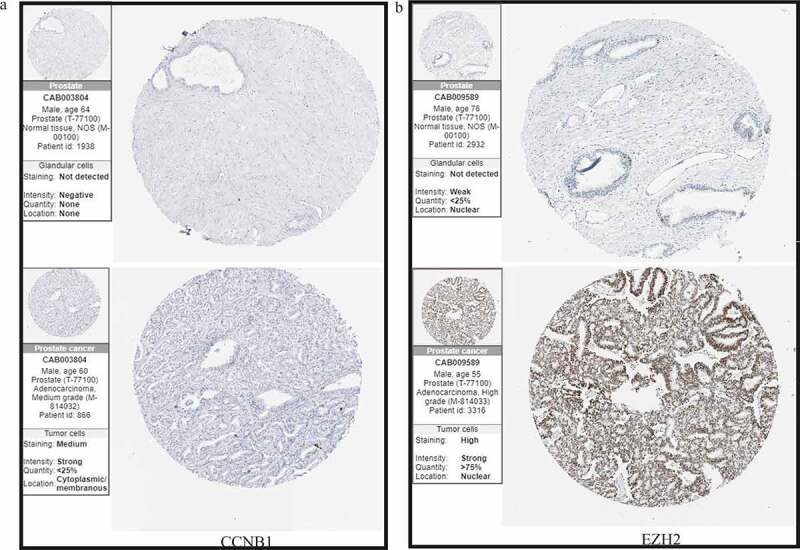
Immunohistochemical analyses confirmed the differential expression of (a) *CCNB1* and (b) *EZH2.*

Immunohistochemistry shown that CCNB1was mainly expressed in cytoplasm and cytoplasmic membrane in PCa cell. However, CCNB1 was failed to be detected in normal tissue. Moreover, EZH2 is highly staining in PCa tissue and located in the nuclear of cancer cell, while it was not detected in normal tissue. These were consistent with the results of mRNA expression obtained from the TCGA, and further verified in transcriptional level (Figure 5).

### 3.6 Correlation between immune infiltration and expression of CCNB1 and EZH2 in PCa

**Figure 6.**
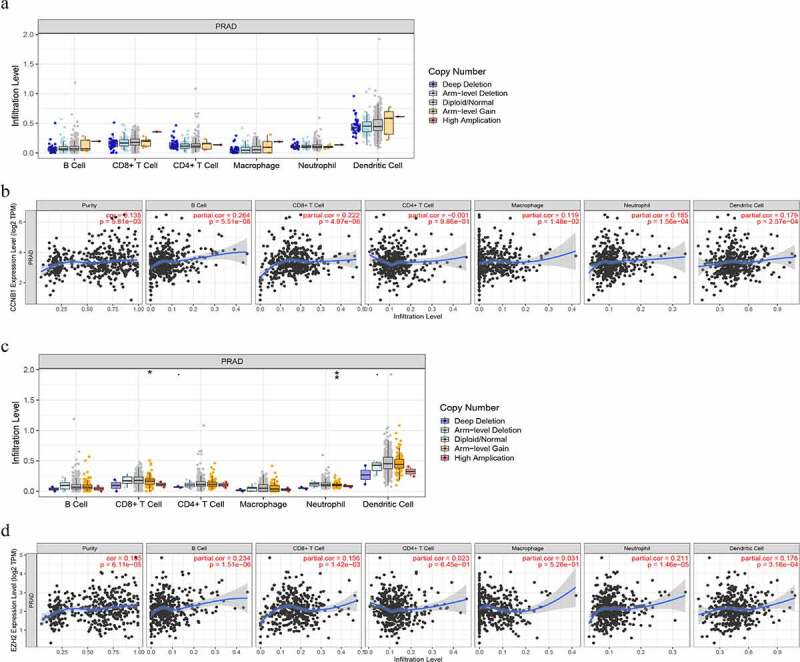
The relationship between immune cell infiltration and altered gene copy numbers of (a) *CCNB1* and (c) *EZH2*; the association between immune cell infiltration level and gene expression of (b) *CCNB1* and (d) *EZH2* (*P* < 0.05)

To further evaluate the relationship between the expression of EZH2, CCNB1 and immune infiltration, we performed following analyses through TIMER. In ‘SCAN’ module analysis indicated that altered gene copy numbers of CCNB1 was not related to immune cell infiltration level. However, the results of ‘Gene’ module indicated that the gene expression of CCNB1 was positively correlated with tumor purity, B cell, CD8 + T cell, macrophage, neutrophil, but not with the immune infiltration level of CD4 + T cell. Moreover, the altered gene copy numbers of EZH2 were associated with CD8 + T cell and neutrophil in PRAD. The gene expression of EZH2 were notably corelated with the immune infiltration level of tumor purity, B cell, CD8 + T cell, and neutrophil (Figure 6).

## 4. Discussion

Docetaxel chemotherapy is a standard treatment for advanced PCa, showing significant symptomatic and survival benefits [11,13]. However, docetaxel resistance in these patients usually occurs after approximately 6 months of systemic therapy. Multiple mechanisms have been reported to be involved in drug resistance, such as limiting intracellular drug concentrations, impaired drug-induced microtubules stability, and neutralizing cytotoxic effects [26–30]. Therefore, targeting drug-resistance and prognosis-related genes has the potential effect to improve chemotherapeutic efficacy and survival. The aim of this study was to identify and analyze the functions of prognostic hub genes, and help understand the molecular mechanisms underlying the development of DRPC and to provide novel gene targets for future studies.

After a systematic search, two microarray datasets were included. In GSE33455 and GSE31635, docetaxel sensitive PCa cell lines were converted to docetaxel-resistant cells to compare the gene expression of sensitive and resistant cells. To improve the reliability of our results, we set significant cutoff value at |log_2_ FC | > 1, adjust *P* value < 0.05 using Benjamini & Hochberg correction to identify DEGs. Finally, 460 DEGs were identified. In order to further investigate the functions of these DEGs, we performed a series of bioinformatic analyses.

The cellular mechanism of drug resistance can be generally divided into two categories: inhibition of chemotherapeutic drug delivery to tumor cells and increased genetic and epigenetic alterations affecting drug sensitivity [31]. GO functional analysis had showed that these DEGs were mainly enriched in the chemotaxis, negative regulation of cell population proliferation, negative regulation of intracellular signal transduction, cellular response to growth factor stimulus, cellular response to lipid, intracellular receptor signaling pathway, regulation of cell adhesion, positive regulation of inflammatory response, regulation of response to cytokine stimulus, lipoprotein metabolic process. Chemotaxis, inflammation and immune cells are closely linked. Chemokines could trigger the chemotactic of immune cells to the site of inflammation [32]. The expression of chemokine receptors (CXCR) is extremely correlated with chemotaxis of immune cell [33]. Previous studies had found that cytotoxic chemotherapy can induce dynamic changes in CXCR expression, which represented a mechanism of acquired chemotherapy resistance [34]. Moreover, activating CXCR4 could contribute to resistance of cancer cells to signal transduction inhibitor and chemotherapy-induced apoptosis [35]. Cell adhesion molecules (CAMs) are in involved in tumor progression, metastasis. Expression of CD44, a member of the CAMs family, could increase drug efflux and lead to drug-resistance [36]. Targeting CAMs has been reported to inhibit metastasis and drug-resistance [37]. Wang et al. addressed that high expression of IFIT3 (an inflammatory-associated gene) is closely related with increased inflammatory response and apoptosis pathways, while knocking down of IFIT3 resulted in reduced chemotherapy resistance of pancreatic ductal adenocarcinoma to paclitaxel [38]. Lactoferrin was demonstrated to have a high binding affinity with lipoprotein, and cloud conjugated to docetaxel to induce tumor targeting effect [39].

KEGG pathway analysis revealed that these DEGs are mainly involved in signaling by Rho GTPases, Miro GTPases and RHOBTB3, interferon signaling, arginine biosynthesis, PI3K-Akt signaling pathway, cytokine-cytokine receptor interaction, MAPK signaling pathway. In xenograft model, Rac1 (one of Rho GTPases), mediates chemotherapy resistance by exerting anti-apoptotic effects [40]. In cancer treatment, interferon (IFN) signaling is essential for optimal chemotherapy response. Exogenous supply of IFN-αβ contributed to chemosensitizing of melanoma cells [41]. Increasing evidences had shown a close relationship between metabolic reprogramming and chemoresistance. Moreover, arginine metabolism was reported to be involved in G6PD induced paclitaxel resistance [42]. PI3K (a lipid kinases) that involved in the regulation of intracellular signaling as well as in the regulation of cellular biological processes. Protein kinase B (AKT), a downstream effector of PI3K, is also involved in multi-drug resistance. It is well demonstrated that PI3K/AKT signaling pathway mediates the process of chemoresistance through multiple pathways, including expression of apoptosis-related proteins, ABC transport, NF-κB, mTOR signaling, etc [43]. In prostate cancer cell lines, inhibition of the MAPK/ERK pathway could contribute to the suppression of cell proliferation and promotes apoptosis, and sensitization to docetaxel treatment [44].

In this study, we identified two prognostic hub genes of DRPC: CCNB1 and EZH2. CCNB1 (cyclin B1), play a pivotal role in mediating cell cycle progression (from G2 phase of cell cycle to mitosis) and metabolism reprogramming in cancer cell [45]. Docetaxel is an m-phase cycle specific drug that causes activation of apoptotic pathways and inhibits cell proliferation [46]. After 24 h treatment of docetaxel, the expression of cyclin B was decreased in PCa cell line by western blotting [47]. Overexpression of CCNB1 could inhibit docetaxel-induced apoptosis and lead to chemotherapy resistance [48].

Enhancer of zeste homolog 2 (EZH2), a family member of polycomb group genes (PcGs), is an important epigenetic regulator in carcinogenesis, involving in cell cycle regulation, apoptosis and anti-senescence [49,50]. Qiu et al. had confirmed that EZH2 was overexpressed in docetaxel resistant PCa cell line by western blotting [51]. Previous studies had also addressed that EZH2 could cause epigenetic silencing of miR-205 and miR-31 to suppress apoptosis of PCa cells induced by docetaxel treatment [52].

Based on the results of functional analyses, we firstly hypothesized that CCNB1 and EZH2 could be involved in chemoresistance through changing the tumor immune microenvironment. Immune cells and cytokines form a complex regulatory network with tumor cells: the tumor immune microenvironment that affect the biological behavior of these cells including: drug resistance [53]. Therefore, we further investigating the relationship between these prognostic hub genes and immune infiltration. We found that the altered gene copy numbers of EZH2 were associated with CD8 + T cell and neutrophil in PRAD, which is consistent with previous literature [54]. Besides, the expression of EZH2 also involved in acquiring M2 phenotype of tumor-associated macrophages (TAMs), activation of dendritic cells (DCs) to mediate epigenetic modification in immunotherapy [55,56]. Our results also shown the notable correlation between the expression of CCNB1 and immune cell infiltration.

The novelty of our work is that we firstly address the correlation between CCNB1, EZH2, and tumor immune microenvironment in docetaxel resistant PCa, and further investigate the relationship between immune cell infiltration and the expression of these two prognostic hub genes.

## 5. Conclusion

In summary, GO and KEGG enrichment analysis confirmed the functions and pathways of these DEGs. In addition, our study identified CCNB1 and EZH2 as prognostic hub genes in docetaxel resistant PCa and further investigated the relationship between immune infiltration and these two genes. The present study will contribute to the understanding of the molecular mechanism development of DRPC and provide new gene targets for future studies.

## Highlights

1. CCNB1 and EZH2 were found to be hub genes in docetaxel-resistance PCa.
2. The expression of CCNB1 and EZH2 are associated with DFS in PCa patients.

(3) The expression of CCNB1 and EZH2 are positively related with multiple immune cells infiltration level.
